# Psychiatric Comorbidity Among Sexual and Gender Minority Youth and Young Adults at Risk for Suicide

**DOI:** 10.1093/eurpub/ckac131.505

**Published:** 2022-10-25

**Authors:** A van der Star, JP Brady, CA Albright, M Gonzales IV, C Garcia Alcaraz, R Cobian Aguilar, A Askew, AJ Blashill, KJ Wells

**Affiliations:** 1 Department of Psychology, San Diego State University, San Diego, CA, USA; 2 Joint Doctoral Program in Clinical Psychology, San Diego State University, UC San Diego, San Diego, CA, USA

## Abstract

**Background:**

Compared with peers, young sexual and gender minorities (SGM) are at a four to seven-fold increased risk of attempting suicide. Prior epidemiological studies, mainly focusing on monomorbid inequalities and without conducting diagnostic clinical interviews, have been unable to report robust data on psychiatric comorbidities among those who attempted suicide. The purpose of this presentation is to describe the presence of current psychiatric comorbidities among SGM youth and young adults at elevated risk for repeat suicide attempts.

**Methods:**

A diverse convenience sample of SGM youth and young adults with a lifetime history of suicide attempts and current suicidal ideation was recruited for an open-phase suicide prevention trial in San Diego, CA. At baseline, participants underwent a 15-module DIAMOND interview for adults or computerized K-SADS for minors, and a battery of self-report questionnaires.

**Results:**

Among the 31 participants (Mage = 22 years [Range: 16, 29]; 100% sexual, 52% gender, and 61% racial/ethnic minority), 27 (87%) participants met criteria for any mood disorder, 24 (77%) for any anxiety disorder, 16 (52%) for any trauma or stress disorder, and 2 (6%) for any psychotic disorder. One (3%) participant did not meet criteria for any psychiatric diagnoses, while five (16%) met criteria for a single and 25 (81%) for multiple diagnoses. The average number of diagnoses was 3.2 (Range: 0, 7). Additionally, 20 (65%) participants met the cut-off for likely ADHD, 20 (65%) for possible borderline personality disorder, and 21 (68%) for likely body dysmorphic disorder, with 11 (35%) within the 90th percentile for reference eating disorder severity.

**Conclusions:**

The degree of psychiatric comorbidities in the sample of SGM youth and young adults at elevated risk for suicide was high. Beside direct suicide risk mitigation efforts, suicide prevention programs that target young SGM with a history of attempts should screen for untreated psychiatric disorders.

**Key messages:**

• LGBTQ+ youth and young adults at elevated risk for repeat suicide attempts experience a high degree of psychiatric comorbity.

• Beyond suicide risk mitigation, LGBTQ+ youth suicide prevention programs should focus on untreated psychiatric comorbidities.

